# Infection of Murine Macrophages by *Salmonella enterica* Serovar Heidelberg Blocks Murine Norovirus Infectivity and Virus-induced Apoptosis

**DOI:** 10.1371/journal.pone.0144911

**Published:** 2015-12-14

**Authors:** Sudhakar S. Agnihothram, Maria D. S. Basco, Lisa Mullis, Steven L. Foley, Mark E. Hart, Kidon Sung, Marli P. Azevedo

**Affiliations:** Division of Microbiology, National Center for Toxicological Research, Food and Drug Administration, Jefferson, Arkansas, United States of America; Robert Koch-Institute, GERMANY

## Abstract

Gastroenteritis caused by bacterial and viral pathogens constitutes a major public health threat in the United States accounting for 35% of hospitalizations. In particular, *Salmonella enterica* and noroviruses cause the majority of gastroenteritis infections, with emergence of sporadic outbreaks and incidence of increased infections. Although mechanisms underlying infections by these pathogens have been individually studied, little is known about the mechanisms regulating co-infection by these pathogens. In this study, we utilized RAW 264.7 murine macrophage cells to investigate the mechanisms governing co-infection with *S*. *enterica* serovar Heidelberg and murine norovirus (MNV). We demonstrate that infection of RAW 264.7 cells with *S*. *enterica* reduces the replication of MNV, in part by blocking virus entry early in the virus life cycle, and inducing antiviral cytokines later in the infection cycle. In particular, bacterial infection prior to, or during MNV infection affected virus entry, whereas MNV entry remained unaltered when the virus infection preceded bacterial invasion. This block in virus entry resulted in reduced virus replication, with the highest impact on replication observed during conditions of co-infection. In contrast, bacterial replication showed a threefold increase in MNV-infected cells, despite the presence of antibiotic in the medium. Most importantly, we present evidence that the infection of MNV-infected macrophages by *S*. *enterica* blocked MNV-induced apoptosis, despite allowing efficient virus replication. This apoptosis blockade was evidenced by reduction in DNA fragmentation and absence of poly-ADP ribose polymerase (PARP), caspase 3 and caspase 9 cleavage events. Our study suggests a novel mechanism of pathogenesis whereby initial co-infection with these pathogens could result in prolonged infection by either of these pathogens or both together.

## Introduction

Co-infection with bacterial and viral pathogens often exacerbates host response and enhances disease severity [1]. Several studies have been carried out to understand the mechanisms regulating the co-infection of bacterial and viral pathogens in the respiratory tract [2–5]; however, few efforts have elucidated molecular mechanisms governing the co-infection of foodborne bacterial and viral pathogens in the gastrointestinal tract. Noroviruses and *Salmonella* strains are major foodborne pathogens in poultry and sea food with pronounced detrimental effects on the gastrointestinal tract [6–8]. Due, in part, to increased *per capita* consumption of poultry and seafood (http://www.cdc.gov/features/dsfoodborneoutbreaks/), there is an augmented risk of co-infection of these pathogens, which may result in severe gastrointestinal disease in humans. Although healthy adults show clearance of norovirus and Salmonella infections in few days, these infections could be life threatening to immunocompromised individuals including young children and elderly populations. A recent study by Gonzalez-Galan et al., showed that in children (<5 years) hospitalized due to acute gastroenteritis illness, norovirus was found to be the most commonly associated pathogen (27% of infected children), followed by rotavirus (21%). Furthermore, 20% of these norovirus infections were associated with a *Salmonella* co-infection [9].

Worldwide, noroviruses infect over 267 million people annually and cause approximately 200,000 deaths, thus they constitute a major public health threat [10–12]. Periodic outbreaks of noroviruses often impose a large burden on global travel, the economy and public health infrastructure, making noroviruses a high priority in terms of addressing unmet medical needs for those who are at risk [13–15]. There are no approved vaccines or therapeutics for noroviruses [16, 17].

Noroviruses (NoV) are non-enveloped viruses, containing a 7.7 Kb-long, single-stranded, positive sense RNA genome [6] and display icosahedral symmetry, with size ranging from 30 to 35 nm in diameter [6]. They belong to the genus *Norovirus* and the family Caliciviridae, which also includes the genera *Sapovirus*, *Vesivirus* and *Lagovirus* [6, 18]. There are seven genotypes designated genogroups I to VII [19, 20], with the human pathogens found mainly in genogroups I, II and IV [6, 20–22].

The molecular mechanisms governing the pathogenesis of the noroviruses are still incompletely understood due to the lack of a robust cell culture and animal model system for human norovirus (HuNoV), that can be utilized for high-throughput analyses [6, 23, 24]. MNV genotype -1 (MNV-1) shows efficient in vitro growth in dendritic cells and macrophages and shows robust replication in mice [24–26]. MNV belongs to genotype V, and has been the most versatile tool to date for understanding norovirus replication, pathogenesis and assessing therapeutic interventions [6, 24, 25]. Murine macrophages, such as the RAW 264.7 cell line, serve as a good host for the MNV-1, in which the virus manifests robust cytopathicity [24]. Although a B cell model exists for HuNov [26] it remains impractical due to the lack of utility for high-throughput analyses and the requirement for Histo Blood Group Antigen (HBGA) expression [26]. Hence, MNV-1 infection of RAW 264.7 cells is an attractive and well-accepted model for studying NoV replication and pathogenesis [23, 24].

In addition to norovirus infections, pathogenic bacteria contribute to 3.6 million food-borne illnesses each year in the USA. Of these pathogens, *Salmonella enterica* serovars alone accounts for about 1 million of these cases, thereby constituting a major public health threat in the USA. Serovar Heidelberg ranks among the top five in causing human salmonellosis in the US [27, 28], with multiple outbreaks and several cases of sporadic cases of gastroenteritis. Approximately 8% of *S*. Heidelberg isolates from human patients originate from the blood, cerebrospinal fluid or joints, which is significantly higher than most other *Salmonella* serotypes [Salmonella Atlas,[29]].Because of this propensity to cause invasive infections, the macrophage model is ideal to evaluate *S*. Heidelberg pathogenicity [30].

Although there is a considerable understanding of pathogenesis mechanisms regulating disease manifestations for norovirus and *Salmonella* individually, little is known about mechanisms that govern co-infection by these pathogens. Murine norovirus have shown to have a lytic replication cycle, where MNV activates caspase-mediated intrinsic pathway of apoptosis to kill infected cells and establish productive infection[23]. On the other hand, *Salmonella* species are invasive pathogens that colonize host cells, prevent cell death and enhance cell survival to established prolonged infection inside host cells [31, 32]. Understanding the molecular mechanisms regulating the co-infection of these pathogens will allow the potential development of therapeutic options that target both pathogens. Hence, we investigated how co-infection by these pathogens affects their replication potential and alters the host response.

## Materials and Methods

### Cell lines, viruses and bacteria

RAW 264.7 murine macrophage cells were obtained from American Type Culture Collection (ATCC), and were grown in Dulbecco’s Modified Eagle’s Medium (DMEM) with 10% fetal bovine serum and 1% Antibiotic-Antimycotic (10,000 units/ml of penicillin, 10,000 μg/ml of streptomycin, and 25 μg/ml of Fungizone® Antimycotic, GIBCO). Cells were seeded on a 6-well plate in complete growth medium one day before infection experiments at a density of 1x10^6^ viable cells/well. Murine Norovirus genotype 1 (MNV-1) was obtained from the American Type Culture Collection (ATCC, Manassas, VA), and was propagated in RAW 264.7 cells. Virus titers were determined by plaque assay on RAW 264.7 cells [33]. *S*. *enterica* serovar Heidelberg isolate 163 [30] was grown in LB broth. A viable cell count, as a function of an optical density standard curve, was generated and used to determine cell inoculum for infecting RAW 264.7 cells. Post-infection bacterial titers were determined by plating serial dilutions on Tryptic Soy Broth with 1.5% agar plates.

### Infection of RAW 264.7 cells with *S*. *enterica* and MNV

Throughout the study, we maintained a ratio of 5 infectious agents (for both *Salmonella* and Murine norovirus) to the target cell, as the multiplicity of infection (MOI). RAW 264.7 cells were infected with a MOI of 5 *Salmonella* cells and/or MNV particles. We reasoned that a MOI of 5 would ensure infection of majority of the cells in the culture, while each cell gets infected with equivalent amount of the bacteria and virus. Experiments constituted three types of infection conditions; a) Prior infection of cells with MNV followed by infection with *S*. *enterica*, referred to as VIR_1_BAC_N_, b) Prior infection with *S*. *enterica* followed by infection with MNV, referred to as BAC_1_VIR_N_ c) Co-infection of cells with MNV and *S*. *enterica* at the same time, referred to as Co-infection. For the condition VIR_1_BAC_N_, RAW 264.7 cells were absorbed with MNV-1 (MOI = 5) for 1h. The virus inoculum was removed and washed with PBS, and DMEM with 5% FBS was added to the cells. Three hours later, the medium was removed. RAW 264.7 cells were then infected with 5 MOI of *S*. *enterica* in serum-free DMEM for 1 h. After incubation, the unbound bacteria were washed three times with PBS. Growth medium with 5% FBS (GIBCO) was then added to the cells. For the condition Co-infection, RAW 264.7 cells were infected with 5 MOI of both bacteria and MNV. After 1h, the inoculum was removed and the cells were washed three times with PBS. DMEM with 5% FBS was then added to the cells. For the condition BAC_1_VIR_N_, RAW cells were infected with 5 MOI of bacteria for 1 h; the medium was removed, and the RAW 264.7 cells were washed three times with PBS. DMEM with 5% FBS was added to the cells, and bacterial growth was allowed for 2h. The medium was removed, and the RAW 264.7 cells were infected with MNV at a MOI of 5 for 1h. After incubation, the cells were washed with PBS and incubated with growth medium. All experimental conditions were assayed with and without gentamicin. In all conditions, supernatants were sampled at 24 h and 48 h to determine infectious virus and 16h, 24h, and 48 h to determine genome copy numbers.

### Viral RNA extraction and analysis of genome copy number

Cell culture supernatants from each experimental condition described were harvested at 16h, 24 h and 48 h post infection to analyze the genome copy number of MNV. Viral RNA was extracted from 115 μl of cell culture supernatant using a MagMAX^TM^ viral RNA isolation kit and the MagMAX™ Express Magnetic Particle Processor (Applied Biosystems/Ambion, Austin, TX). Extracted RNA samples were either analyzed immediately or stored at −80°C until use. RNA samples were analyzed for norovirus by a real-time one-step RT-PCR, using the Rotor-Gene Multiplex RT-PCR kit (Qiagen, Valencia, CA) according to the manufacturer’s instructions. The reactions were carried out in duplicate, using primers and a Cy5-labeled fluorescent probe (Integrated DNA Technology, Coralville, IA) targeting ORF2 region (position 6520 to 6645). MNV RNA was used to generate a standard curve and 18S RNA (Applied Biosystems, Foster City, CA) was used as an internal control (16). RNA from mock-infected (no virus infection) RAW 264.7 cell supernatant was used as negative control and RNAse-free water was used as a non-template control. Data were transformed in gene copy number using a standard curve generated from serial dilutions of virus stock preparations previously tittered by plaque assay. The detection limit of the assay was determined to be 2 RNA copies. Amplification was detected using a Rotor Gene Q6-plex machine from Qiagen. Raw data were analyzed using Rotor-Gene™ software. Briefly, 5 μl of RNA was transferred to a Qiagen Rotor-Gene strip tube containing 10 μl of 2 × Rotor-Gene Multiplex RT-PCR master mix, 800 nM of each norovirus primer [34] MNVF (5’-TGCAAGCTCTACAACGAAGG-3’), MNVR (5’-CACAGAGGCCAATTGGTAAA-3’), 200 nM of probe MNVP (5’-Cy5-CCT TCC GCA CCG ATG GCA TG-IBRQ-3’), and 0.2 μl of Rotor-Gene RT mix. The thermal cycling conditions consisted of reverse transcription at 50°C for 30 min, 95°C for 10 min, and then 50 cycles of 94°C for 30 sec and 48°C for 60 sec.

### Immunofluorescence experiments for caspase-3 and cell death assays

RAW 264.7 cells under the previously described experimental conditions were analyzed for the presence of activated caspase 3, using Cell Event Caspase 3 Detection reagent (Molecular Probes, Eugene, OR) according to the manufacturer’s instructions. Briefly, 2 drops of caspase 3 detection reagent was added per ml of the growth medium for cells under each treatment conditions at 24h post infection [23]. This reagent contains a non-fluorogenic caspase 3 substrate (DEVD) which, upon cleavage by activated caspase 3, produces a bright fluorogenic response when excited at 488 nm. Cells with the dye were incubated at 25°C for 30 min, and images were taken at 10x magnification upon excitation at 488 nm using an inverted Nikon Fluorescence microscope. To label dead cells, Dead Red Dye (Molecular Probes, Eugene, OR) was used. RAW 264.7 cells under various experimental treatment conditions were analyzed for cell death at 48 h post infection. Briefly, Dead Red Dye was added to the medium according to the manufacturer’s instructions, incubated at 20–25°C for 15 min, and imaged at 10 x magnification using a TRITC excitation filter and an inverted Nikon Fluorescence microscope.

### Quantification of MNV bound to RAW 264.7 cells

RAW 264.7 cells were infected either with MNV at a MOI of 5 for 1h (Virus alone or V_1_B_N_), or co-infected with 5 MOI of *Salmonella* (co-infection) for 1h, or infected first with 5 MOI of *Salmonella* followed by a 5 MOI of MNV infection (B_1_V_N_) at 2 hours post bacterial invasion. Mock infection of RAW 264.7 cells included media control. In all conditions, cells were fixed with 2% cold paraformaldehyde (Sigma Aldrich, St. Louis, MO) 1h after virus infection. Cells were blocked with block solution (PBS 5% FBS), and were stained using 1:300 dilution of 5C4 antibody, kindly provided by Dr. Christiane Wobus (University of Michigan, Ann Harbor) [35] in block solution for 1 hour at 4°C. Unbound antibody was washed with the block solution. Bound antibody was detected using an anti-mouse FITC (Molecular Probes, Eugene, OR), and imaged using a 488 nm filter in an inverted fluorescence NIKON microscope. For quantification, images of cells from 8–10 fields were captured randomly using 488 nm filter, along with a respective bright field image. Percent of virus positive cells in each field was calculated and averaged under each condition, and represented as a histogram.

### Plaque assay for MNV

Viral titers were determined by a plaque assay using RAW 264.7 cells as described previously [33]. Briefly, virus supernatants were serially diluted in PBS, and the dilutions were inoculated on a confluent monolayer of RAW 264.7 macrophages and allowed to incubate at room temperature for 1 h. Viral inocula were removed and cells were washed twice with PBS. A 1.5% agarose overlay in DMEM was added to the cells and incubated at 37°C. Plaques were counted at 48–72 h post incubation. Virus titers were estimated by multiplying the mean of plaque numbers in at least two wells times the reciprocal of the lowest virus dilution with visible plaques.

### Determination of bacterial counts

RAW 264.7 cells described in each condition were lysed in PBS containing 1% Triton X-100. Cell lysates were serially diluted in PBS and plated on tryptic soy agar (Difco) plates. The plates were incubated for 24 h and bacterial colonies for each dilution were counted in triplicate. Values were presented as colony forming units per milliliter (cfu/ml).

### DNA fragmentation

Chromosomal DNA was isolated using DNA Zol (Molecular Sciences) according to the manufacturer’s instructions, and was analyzed using 1.5% agarose gel electrophoresis at 90 volts for 1 h, followed by exposure.

### Analysis of cytopathic effects in cells infected with MNV and bacteria

Cells in each condition were monitored for cytopathic effects (cell detachment, rounding and floating in medium) using bright-field light microscopy, and images were taken using a 10 X objective in a Nikon inverted light microscope.

### Immunoblotting

Cell culture supernatants were removed from RAW 264.7 cells from the various treatment conditions at 24 h post infection. Cell monolayers were washed with cold PBS (pH 7.4) once, after which the cells were lysed in lysis buffer (1% Triton X-100, 50 mM Tris, 150 mM EDTA), containing a protease inhibitor cocktail (Zymogen). Cells were vortexed to ensure complete lysis, and cell debris was pelleted by centrifuged at 13,000 x *g* at 4°C for 10 mins. Equivalent amounts of protein were loaded to SDS PAGE TGX 4–15% gradient gels (Bio-Rad) and resolved. Proteins were transferred nitrocellulose membranes using a Bio-Rad Western Transfer apparatus. Primary antibody staining was performed for 14 h at 4°Cin blocking buffer (0.1% Tween 20 in Tris Buffered Saline). Secondary antibody staining and chemiluminiscence detection (using Bio-Rad Clarity Western Substrate) was performed the for 1h. Antibodies to cleaved caspase 9, caspase 3 and PARP were obtained from Cell Signaling Technologies (Danvers, MA), and horse-radish peroxidase-linked secondary anti-rabbit and anti-mouse antibodies were purchased from Thermo Fischer Scientific (Waltham, MA).

### Enzyme linked immunosorbent assay (ELISA)

Cell culture supernatants collected at 48h post infection were aliquoted into 200 μL volumes and frozen at -80°C. An ELISA test was conducted for IFN-γ, IL-6 and TNF-α according to the manufacturer’s instructions (R&D Biosystems). Briefly, wells of Nunc Maxisorp 96-well plates were coated with anti-mouse IL-6 (2 μg/ml), anti-mouse IFN-γ (4 μg/ml) or anti-mouse TNF-α (0.8 μg/ml) (R&D BioSystems) overnight at room temperature. Before use, the plates were blocked with PBS containing 1% bovine serum albumin fraction V (BSA, Sigma) for 2 h at room temperature. Cell culture supernatant samples were added to the wells in a volume of 100 μl and the control samples were diluted in PBS 1% BSA. After the 2 h incubation at room temperature, wells were washed 4 times with PBS containing 0.05% Tween 20. The addition of the biotinylated monoclonal antibodies for each of the cytokines (IL-6 150ng/ml; IFN-γ 300ng/ml; TNF-α 50ng/ml) were added and incubated for 2 h at room temperature. Horseradish peroxidase-conjugated streptavidin (R&D Biosystems) was added according to the manufacturer’s recommendations and incubated for 30 min at room temperature. Standard curves were generated using purified recombinant IL-6, IFN–γ and TNF-α according to the manufacturer’s recommendations (R&D BioSystems) using MaxPro generated 4-parameter curve-fit for each cytokine.

### Statistical analyses

All the data were plotted, analyzed and statistical analysis was performed using Graph Pad Prism software. Two-tailed T-tests were used to analyze statistical significance between the experimental groups.

## Results

### *S*. *enterica* infection reduces replication of Murine Norovirus in RAW 264.7 cells

**Fig 1:**
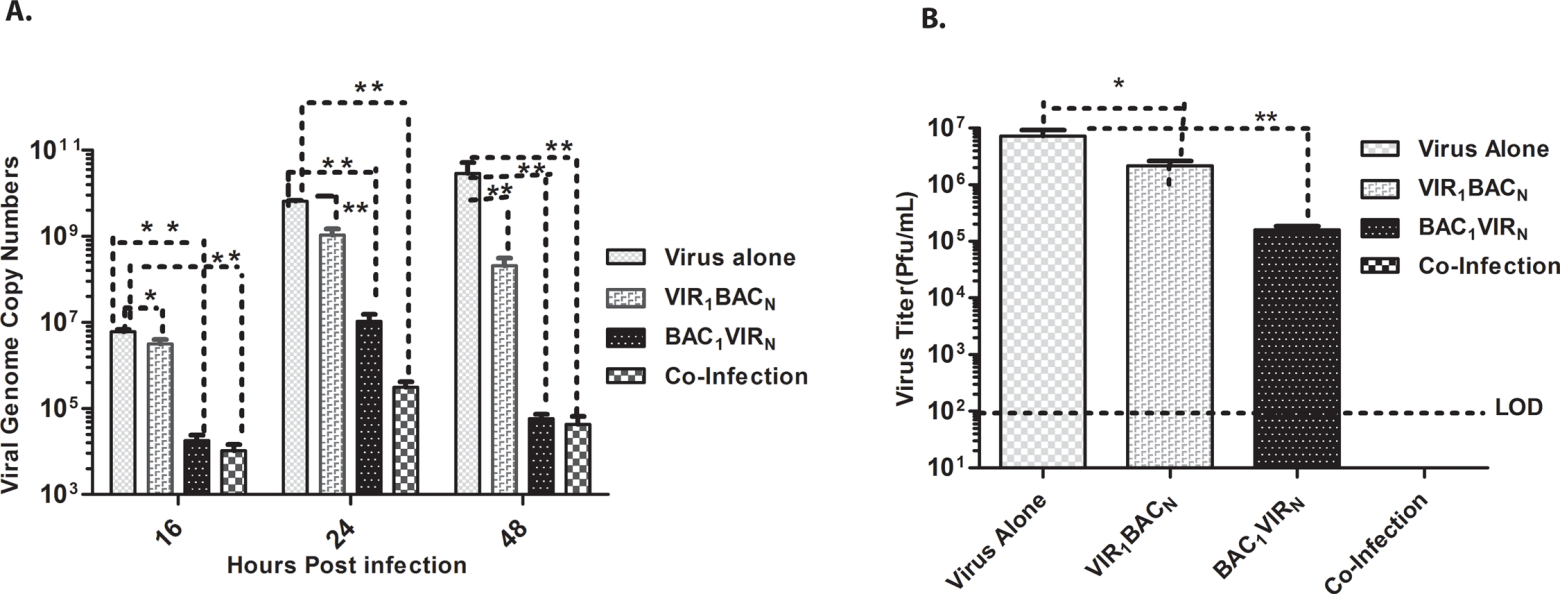
MNV replication is reduced in *S*. *enterica* infected cells. A. Viral genome copy numbers from cell culture supernatants from different treatment conditions (n = 3), harvested at indicated time points as measured by real time RT-PCR; B. Virus titers as measured by plaque assay from cell culture supernatants harvested at 24 h post infection from different treatment conditions. ** P<0.005, * P<0.05, as determined by Student`s t- test. Results represent the mean of two independent experiments, error bars indicate SEM.

To analyze whether exposure of RAW 264.7 cells to *S*. *enterica* affects replication of MNV, we analyzed the replication of MNV under three different conditions: pre-exposure (BAC_1_VIR_N_) of the RAW 264.7 cells to *S*. *enterica*, followed by infection with MNV; co-infection of RAW 264.7 cells with bacteria and the virus simultaneously; or post-exposure (VIR_1_BAC_N_), where the cells were initially infected with MNV and then *S*. *enterica*. Pre-exposure of macrophages to bacteria caused a time dependent decrease in viral replication when compared to virus alone condition as shown by ~4- and 6 -log reduction in genome copy numbers at 16 and 48 h respectively, and a 2 log reduction in infectious virus titers at 24 h (Fig 1A and 1B). While co-infection resulted in drastic reduction of infectious virus titers to below the limits of detection at 24 h (Fig 1B), the genome copy numbers showed a ~4- and 6 -log reduction at 16 and 48 h respectively, when compared to virus alone condition (Fig 1A). Infection of cells with MNV at 3 h after bacterial inoculation allowed efficient virus replication (infectious virus titers at 24 h was ~4x10^6^pfu/ml, and viral genome copy numbers showed 0.5 log reduction at 16 h and 2 log reduction at 48 hours when compared to virus alone condition) (Fig 1A and 1B).

### Replication of *S*. *enterica* is increased upon infection with MNV

**Fig 2:**
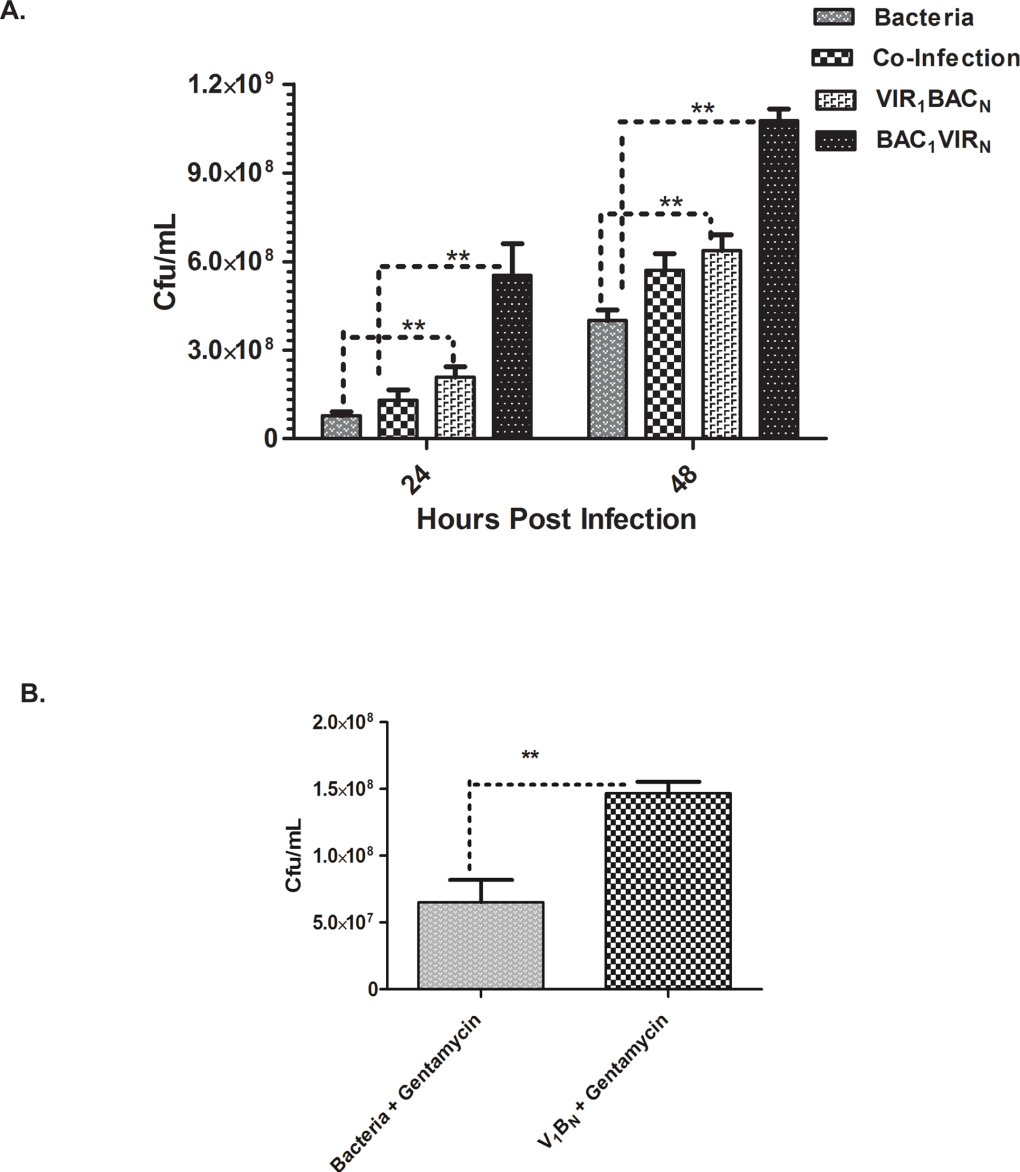
*S*. *enterica* infection is increased in MNV-infected macrophages. A. Bacterial counts from cell lysates harvested at indicated time points under different treatment conditions (n = 3) at indicated time points. ** P<0.05 as determined by Student`s t-test. Results represent the mean of two independent experiments; error bars indicate SEM. B. Bacterial counts showing increase in bacterial titer due to prior virus infection at 24 h post infection in the presence of 100μg/ml gentamicin. ** P<0.05 as determined by Student’s t-test, error bars indicate SEM.

To assess whether bacterial replication was affected by the presence of MNV, we analyzed the bacterial numbers in RAW 264.7 cells harvested at 24 and 48 h post infection. Time-matched triplicate controls where RAW 264.7 cells were infected with bacteria alone, served for comparison for baseline bacterial titers. Replication of bacteria was enhanced up to ~3 fold at 24 and 48 h in cells first infected with bacteria followed by infection with MNV (Fig 2A, BAC_1_VIR_N_). Interestingly, prior exposure of cells to MNV (VIR_1_BAC_N_) or co-infection of *Salmonella* with MNV enhanced bacterial replication up to ~2 fold at 24 and 48 h, when compared to the control bacterial titers in cells infected with MNV alone (Fig 2A). The virus-induced increase in bacterial replication was also evident with the presence of 100μg/ml of gentamicin in the media (Fig 2B).

### MNV induced cytopathic effect (CPE) is blocked in cells infected with *S*. *enterica*

**Fig 3:**
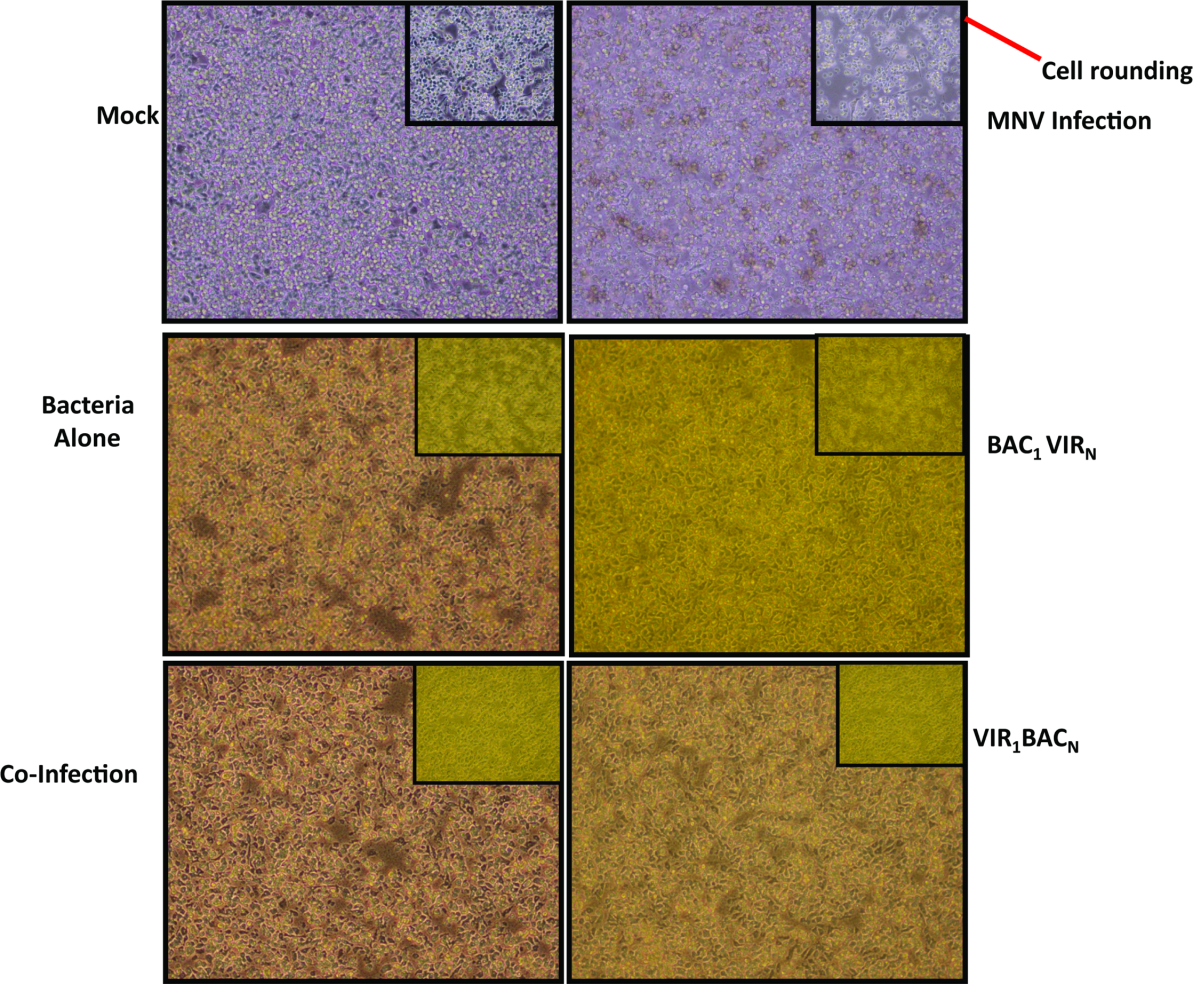
MNV-induced cytopathicity is reduced in *S*. *enterica* infected cells. Representative white field images (10 X) of RAW 264.7 cells taken at 48 h post infection under indicated treatment conditions. The 20 X magnification insert is included in each picture. Note that virus-infected cells show cytopathic effect (cell rounding and clearance of monolayer, shown by red arrow) which is clearly evident in the 20 X magnification insert as indicated. Intact monolayer is seen in other conditions.

MNV has been shown to cause rapid CPE at 48 h post infection [23]. Similar observations were documented in MNV-infected RAW 264.7 cells in our laboratory under the same experimental conditions. Since a drastic reduction of virus replication was observed in cells infected first or coinfected with the bacterium, the next step was to determine whether the virus-induced CPE was also reduced. At 48h post infection, all cells were imaged under the inverted light microscope. As expected, monolayers showed rapid cell rounding and cytopathicity in cells infected with MNV (Fig 3, 20 X insert in MNV infection). In contrast, all of the cell monolayers engorged with bacteria were still viable (Fig 3, center and bottom panels). A close look revealed an elongated morphology, indicating that these macrophage cells might be activated due to the infection by *S*. *enterica*, as RAW 264.7 cells are reported to exhibit elongated morphology upon activation by immunostimulation [36]. These results clearly demonstrated that bacteria were blocking virus-induced CPE. In the infection condition VIR_1_BAC_N_, where MNV replicated to about 10^6^ pfu/ml (Fig 1B), a reduction in CPE was evident (Fig 3, VIR_1_BAC_N_) indicating that the bacteria inhibited virus-induced cell death, not just virus replication.

**Fig 4:**
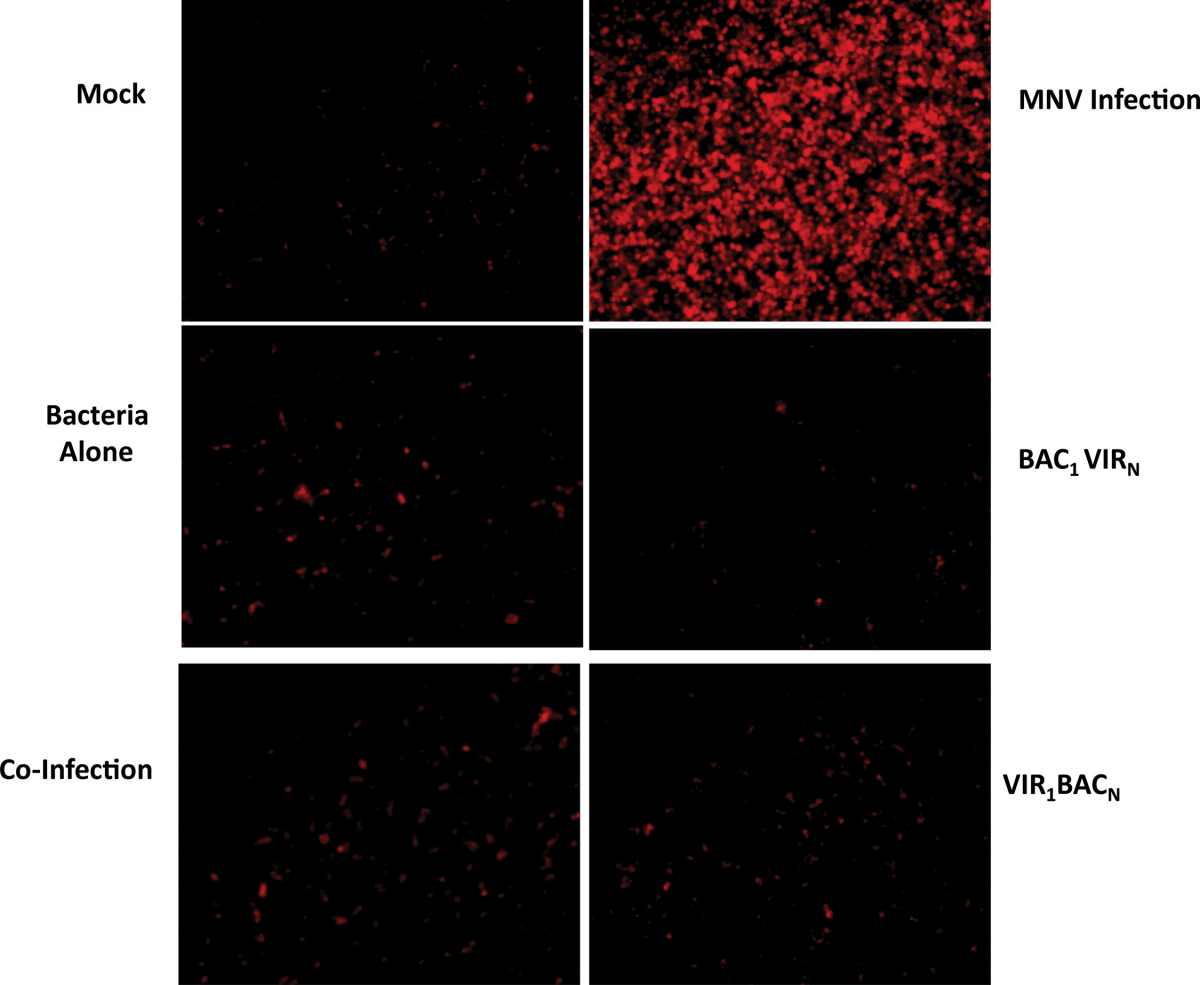
MNV-induced cell death is reduced in *S*. *enterica* infected cells. Representative images of cells stained with Dead Red Dye from Molecular Probes at 48 h post infection, and captured by a TRITC filter using an inverted fluorescence microscope. Red cells indicate cells with compromised membranes. Note the high number of red cells with MNV infection, and drastic reduction in other conditions.

The inhibition of MNV- induced cell death in *S*. *enterica* infected cells at 48 h post-infection was further confirmed by Dead Red Dye staining. The majority of the cells infected with MNV alone stained bright red, indicating the ongoing cell death (Fig 4). Conversely, the numbers of dead cells were drastically reduced in all other conditions where there was active *S*. *enterica* replication, which demonstrated that inhibition of cytopathic effect in MNV infected RAW 264.7 cells resulted in reduced cell death.

### MNV-induced apoptosis is inhibited in cells infected with *S*. *enterica*

**Fig 5:**
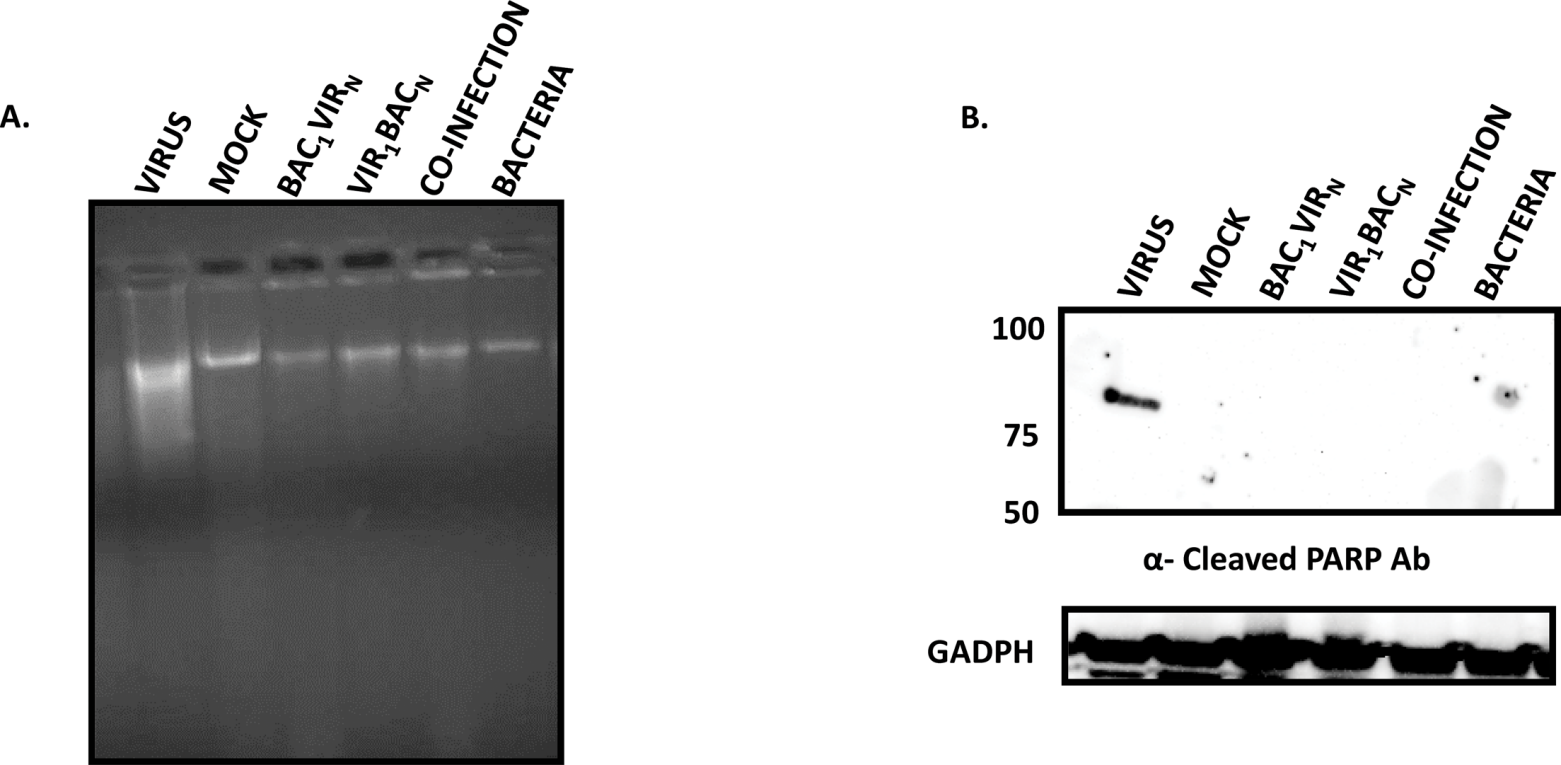
MNV-induced apoptosis inhibited in *S*. *enterica* infected cells. A. Chromosomal DNA from RAW cells under different treatment conditions was isolated and analyzed by agarose gel electrophoresis. Note that DNA is fragmented, as shown by the smear, whereas intact genomic DNA is observed in all other conditions; B. PARP cleavage inhibition in *S*. *enterica* infected cells, shown by western blot analysis of cell lysates harvested at 24 h post infection under indicated conditions and stained with anti-PARP antibody. GADPH loading control is shown below.

MNV has been observed to cause caspase-dependent apoptosis through the mitochondrial pathway, which also involves fragmentation of the cellular DNA [23]. The absence of CPE prompted us to examine whether apoptosis was inhibited in *S*. *enterica*-infected cells. To investigate this, we initially analyzed the integrity of genomic DNA isolated from cells at 48 h post infection from all the experimental conditions, using agarose gel electrophoresis. DNA was smeared in cells infected with MNV, as reported previously [23], whereas intact DNA was observed from cells with mock infections, and other infection conditions with the bacteria (Fig 5A). Interestingly, DNA from cells infected first with virus (VIR_1_BAC_N_) was still intact despite the presence of high viral titers (VIR_1_BAC_N_, Fig 5A), as MNV replication has been shown to cause DNA fragmentation due to apoptosis [23]. DNA from cells under all other treatment conditions was found to be intact, similar to DNA from mock-infected cells (Fig 5A).

### Poly ADP Ribose Polymerase (PARP) cleavage is inhibited in cells infected with *S*. *enterica* and MNV

PARP cleavage is a well-studied marker for induction of apoptosis induced by DNA damaging agents [37]. PARP cleavage signals the beginning of apoptosis, which then results in further downstream degradation of DNA [37]. MNV induces cleavage of PARP starting at 16 h post infection [23]. We investigated whether the inhibition of apoptosis in MNV infected cells by *S*. *enterica* also resulted in inhibition of PARP cleavage. Western blot analysis revealed clear detection of PARP at ~80 kDa in MNV infected cells at 24 h post infection, indicating progression of apoptosis (Fig 5B). PARP cleavage was not observed in cells infected with *S*. *enterica* under other experimental conditions. In particular, pre-exposure of cells to MNV followed by bacterial infection (VIR_1_BAC_N_), which had increasing virus titers, showed a complete absence of PARP cleavage, indicating inhibition of virus-induced apoptosis by *S*. *enterica*.

### Caspase 3 and 9 cleavage events are inhibited in cells infected with *S*. *enterica*

**Fig 6:**
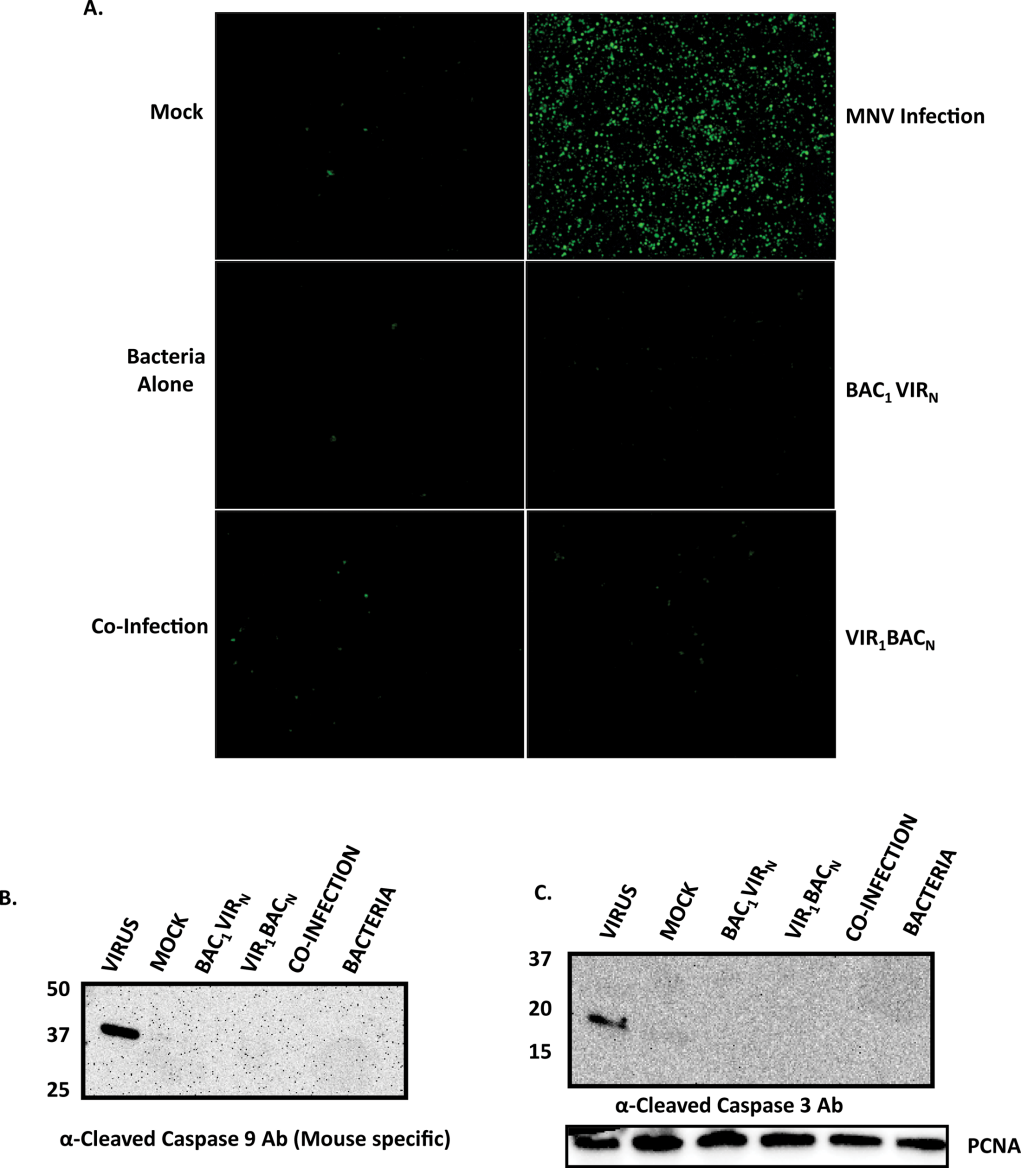
*S*. *enterica* infection inhibits MNV induced caspase 9 and caspase 3 cleavage. A. Representative 10 X images of cells under different experimental conditions as labeled by Cell Event Caspase-3 Dye at 24 h post infection. Note the abundance of green cells in MNV infected monolayers, while green cells are drastically reduced in other conditions; B. Western blot analysis of representative cell lysates harvested at 24 h post infection and stained with mouse specific caspase 9 antibody (A) and caspase 3 antibody (B). Molecular weights in kDA are indicated on the side. Proliferating Cell Nuclear Antigen (PCNA) loading control is shown below.

MNV has been shown to induce apoptosis in RAW 264.7 cells through the caspase 3 and caspase 9 pathways [23]. Results from our current study indicate a clear inhibition of MNV-induced apoptosis by *Salmonella enterica* as evidenced by the absence of DNA fragmentation and PARP cleavage (Fig 5A and 5B). At a molecular level, progression of apoptosis involves cleavage of PARP by activated caspase 9 and caspase 3 [38–41], which occurs in MNV infected RAW 264.7 macrophages [23]. Therefore, we hypothesized that absence of PARP cleavage might represent an inhibition of the upstream cleavage events of caspase 3 and caspase 9. To evaluate this hypothesis, cells under various experimental conditions were labelled at 24 h post infection with a fluorescently labeled substrate for caspase-3 (Cell Event Caspase 3) that exhibits green fluorescence in cells upon the cleavage of the substrate by activated caspase 3 [42]. The majority of the cells in the MNV infected population showed enhanced green fluorescence (Fig 6A), indicating the presence of cleaved caspase 3 during virus infection. Reduction of cells with green fluorescence was observed in all other experimental conditions where there was bacterial replication. These observations clearly indicated the inhibition of caspase-3 cleavage in cells infected with *S*. *enterica* (Fig 6A). These results were further confirmed using western blotting of the cell lysates using an antibody directed to the cleaved caspase 3, demonstrating the absence of cleaved caspase 3 in all experimental conditions, except when RAW 264.7 cells were infected with MNV alone (Fig 6C). In addition, we detected the presence of cleaved caspase 9 at 37 kDa in MNV infected cells, but not in cells under other experimental conditions (Fig 6B). These results demonstrated the blockade of caspase 9 and caspase 3 cleavage by *S*. *enterica*, despite allowing MNV replication.

### Upregulation of inflammatory and antiviral cytokines in cells infected with *S*. *enterica* may correspond with observed reduction in virus titers

**Fig 7:**
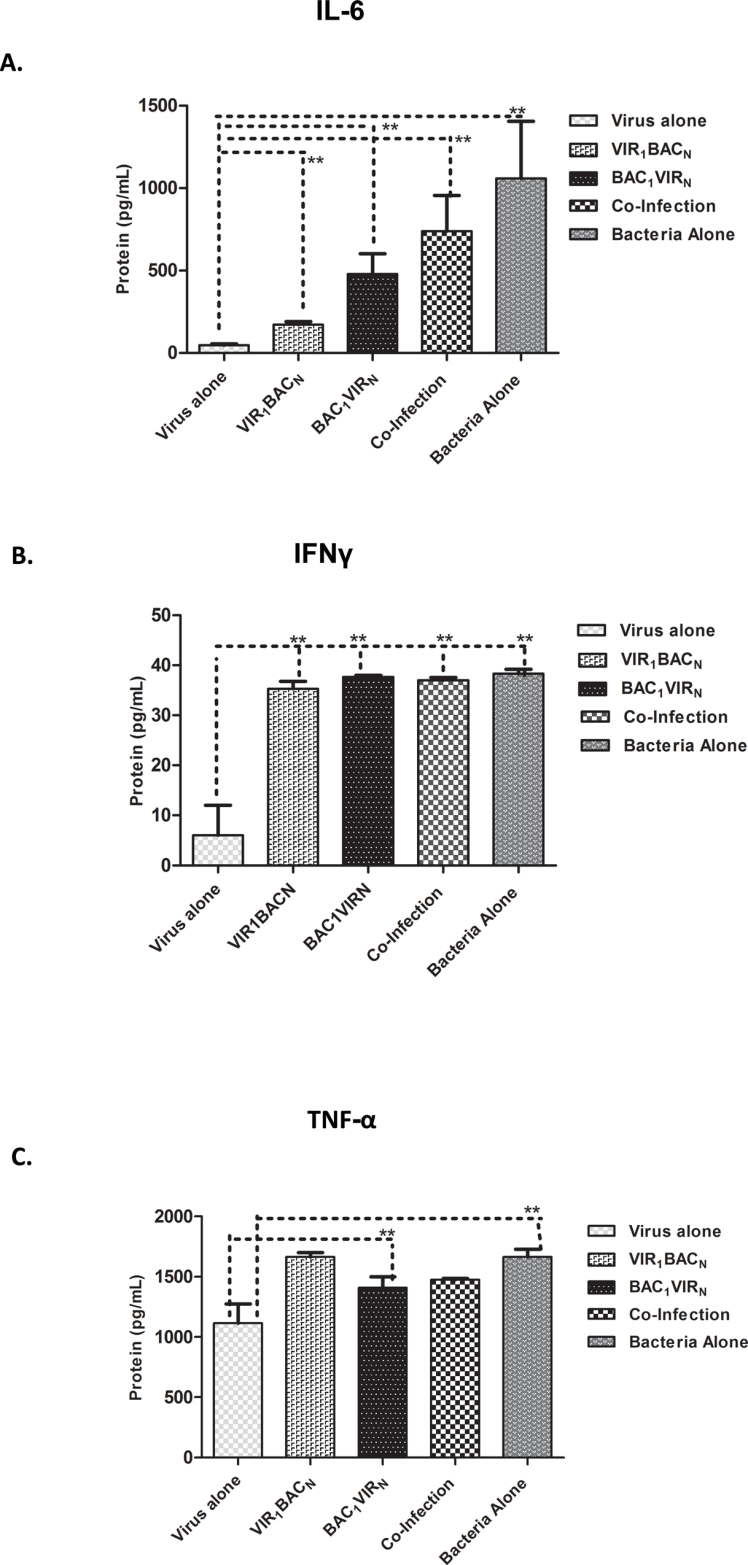
ELISA results showing elevation of cytokines. Protein content in pg/ml of the indicated cytokines as measured by ELISA, from cell culture supernatants harvested at 48 h post infection. A. IL6; B. IFNγ; C. TNFα. ** P<0.05, as measured by Student’s t-test.

The study results clearly indicated that *S*. *enterica* infection modified the host RAW 264.7 cells at a molecular level leading to inhibition of apoptosis. In addition, the presence of the bacteria in macrophages caused a drastic reduction in virus replication. This could occur due to the induction of the pro-inflammatory and antiviral cytokines that might be upregulated upon infection with the bacteria alone or both pathogens. Bacterial replication could trigger a proinflammatory environment leading to the up regulation of cytokines resulting in reduction of virus replication. To investigate the roles of cytokines and chemokines in blocking viral replication in *Salmonella* infected cells, the presence of cytokines in cell culture supernatants of cells was analyzed following various infection conditions at 48 h post infection. We observed clear differences with cytokines including Interleukin 6 (IL6), Interferon Gamma (IFNγ), and Tumor Necrosis Factor Alpha (TNFα). There was an inverse correlation of IL6 levels with virus replication (Fig 7A). While a low level of IL6 was observed in cells infected MNV alone, IL6 levels increased in other conditions (Fig 7A). IFNγ and TNFα were at lower levels in cells infected with MNV alone, but the presence of bacteria lead to increased production of these cytokines (Fig 7B & 7C). These results suggested that the bacterial replication stimulated secretion of proinflammatory cytokines, which might alter viral replication through mechanisms that still need to be determined.

### Presence of *Salmonella* prior to or during MNV infection affects binding of MNV to macrophages

**Fig 8:**
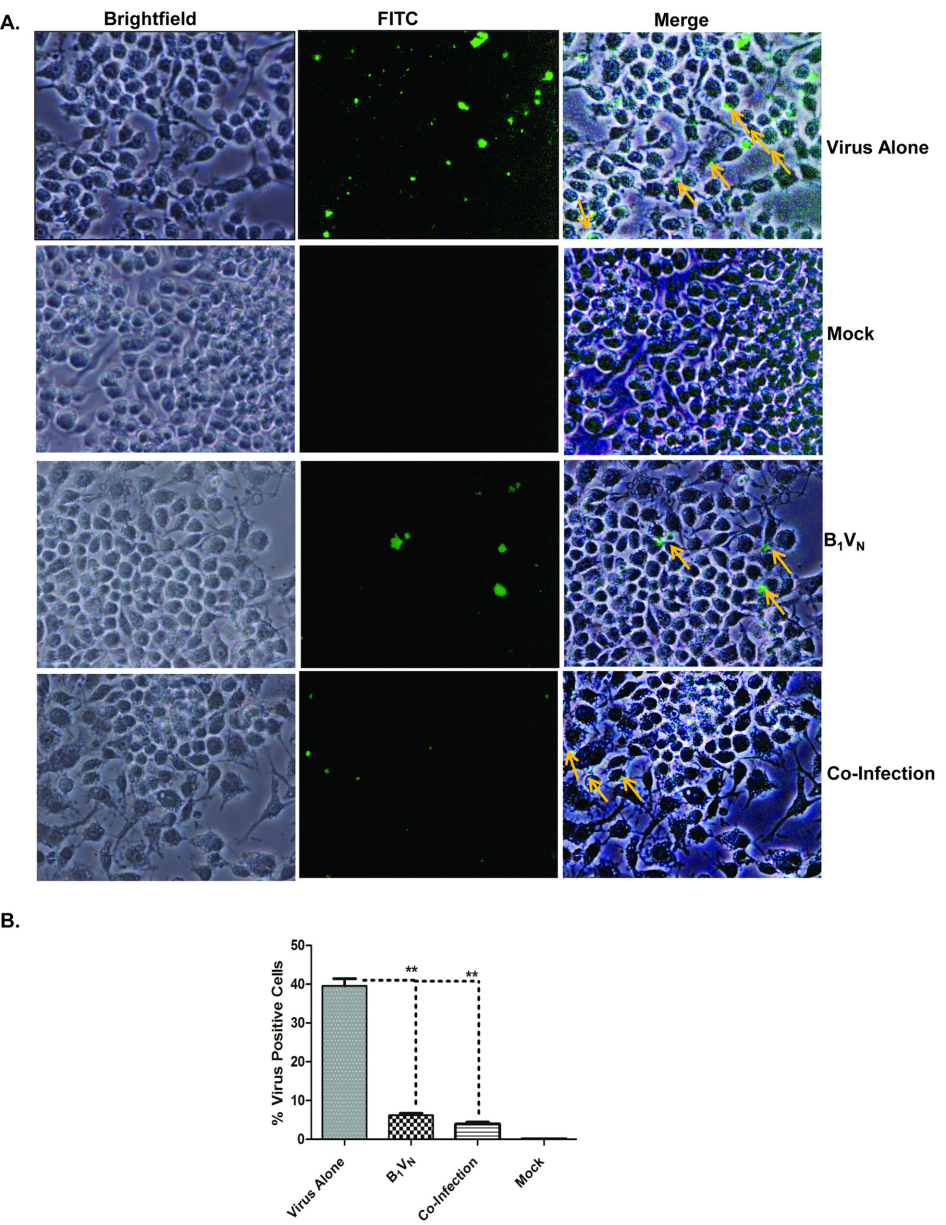
Cell Surface Staining Reveals Bacteria Inhibiting Virus Binding to RAW 264.7 Macrophages. A. 40x Images of cells under indicated experimental conditions stained with 5C4 antibody and imaged through 488 nm filter or bright field. Cells with virus bound fluoresce as green, and antibody-bound virus on cell surface is indicated by orange arrows in the merged image. Note the drastic reduction in virus-positive cells in B_1_V_N_ condition and Co-infection. B. 8–10 fields were imaged randomly in bright field and with 488 nm filter in each condition, and percent of virus positive cells was calculated for each condition and plotted as a histogram.**P<0.05, as determined by Student’s t-test, error bars indicate SEM.

Because virus replication was reduced when bacterial inoculation preceded occured simultaneously with the virus, we investigated whether *Salmonella* affects MNV entry under the various experimental conditions. Using 5C4, an antibody to MNV capsid [35] we performed indirect immunofluorescence, and quantified percentages of virus-positive cells. The antibody exhibited high specificity as shown by the absence of staining in mock infected cells (Fig 8A). Higher number of virus-bound cells was detected when cells were first infected with virus, whereas there was a drastic reduction in number of virus-bound cells under conditions of co-infection or when the bacterial infection preceded virus infection (B_1_V_N_. Fig 8A). Quantification revealed that while 45% of cells were positive for cell-surface bound virus when infected alone, only 8–10% and 5% of cells were found to be virus-positive under conditions of co-infection or BAC_1_V_N_ (Fig 8B), indicating that the presence of bacteria prior to or during virus infection affects virus binding to RAW 264.7 cells.

## Discussion

Foodborne gastroenteritis is a continued public health threat. *Salmonella* and noroviruses individually contribute to about 35% of the foodborne illness throughout the world, and surveillance reports indicate an increase prevalence of norovirus co-infection in patients suffering from gastroenteritis caused by *S*. *enterica* [9]. Therefore, understanding the mechanisms regulating pathogenesis during co-infection of these pathogens is important from a public health prospective.

To this end, this study examined the mechanisms that regulate co-infection of the *Salmonella* and noroviruses. We chose *Salmonella enterica* serovar Heidelberg and murine norovirus for our studies. Both of these organisms infect RAW 264.7 cells and replicate efficiently in this cell line [24, 30]. Peak MNV viral titers reached 10^7^ pfu/ml (Fig 1B), and the bacterial counts reaching 10^8^ cfu/ml (Fig 2) at 24 hours post infection, indicating that RAW 264.7 cells were a suitable host for studying host pathogen interactions for both of these pathogens [23, 24].

When the effect of bacterial infection on MNV replication in RAW 264.7 cells was assessed, the results indicated that MNV replication was abrogated early (16 hours post infection) in cells co-infected with bacteria or cells pre-exposed to bacteria before viral infection (BAC_1_VIR_N_), while cells first exposed to virus infection (VIR_1_BAC_N_) showed a moderate amount of virus replication (Fig 1A and 1B). A similar pattern was observed at 24 and 48 h post infection, suggesting that fewer virus particles might have gained entry into the cells under these conditions. Quantification of virus-bound cells after 1h post-virus infection clearly revealed that presence of bacteria prior to, or during virus infection drastically impairs the ability of virus to bind to cells, which might result in abrogated virus entry. Murine noroviruses have been shown to bind ganglioside-linked terminal sialic acid moieties on the surface of murine macrophages for cell attachment and entry [45]. The block in MNV entry by the bacteria could occur as result of engagement of virus receptor by bacterial surface adhesion molecules, or *P*athogen *A*ssociated *M*olecular *P*atterns (PAMPs) found on the surface of *S*. *enterica*, while the cells previously exposed to the MNV (VIR_1_BAC_N_-condition) might have allowed for virus entry and subsequent replication. Such observations have been previously reported for Dengue Virus infection of human monocytes/macrophages, where the bacterial lipopolysaccharide (LPS) engages the CD14 receptor and blocks Dengue Virus entry and replication if the LPS is added to cells before Dengue Virus infection, but the virus is able to replicate efficiently if the virus infection precedes LPS exposure [43]. Similar observations have also been reported for HIV-1 and Lymphocytic Choriomeningitis Virus (LCMV) [44–46]. Further studies are needed to evaluate this hypothesis.

While the entry block observed in co-infection condition might have resulted in drastic reduction in MNV replication, MNV still replicated to 10^5^ pfu at 24 h post infection when virus infection followed bacterial invasion (B_1_V_N_). This difference could be due to the continued presence of high titers of bacteria in the supernatant during MNV infection under co-infection conditions. While the presence of extracellular bacteria did cause a drastic reduction in virus binding to cells (Mean = 3.9% virus-positive cells, Fig 8B), parallel bacterial invasion during entry of virus particles that had bound to cell surface might have an effect on early events in virus life cycle including uncoating, genome transcription and replication. These alterations might drastically impact the virus-life cycle and formation of infectious progeny virus, as shown by absence of detectable virus titers at 24h post infection. Under BAC_1_V_N_ conditions, although initial entry block was observed (Mean = 6.3% virus-positive cells, Fig 8B), absence of parallel bacterial invasion might allow for efficient execution of early events in virus-life cycle, resulting in a moderate impact on the virus-life cycle with infectious progeny virions being formed. MNV-induced apoptosis enables the virus release out of the cell which might result in enhanced cell to cell spread and increased replication. Apoptosis inhibition by Salmonella could also result in observed reduction in virus replication.

In our study, there was an increase in bacterial replication in cells infected with MNV (Fig 2); which may be due to the upregulation or enhancement of host factors that facilitate bacterial replication in RAW 264.7 cells. This finding could also involve upregulation of cell surface receptors that might enhance bacterial entry and cell-to-cell spread. In cases of secondary bacterial pneumonia, bacterial replication is primed by viruses that infect the respiratory tract, including Influenza viruses, Rhinoviruses and Respiratory Syncytial Viruses [2–4, 47]. Prior viral infection primes the respiratory epithelial cells to upregulate chemokines and cell surface receptors that enhance adhesion of *Staph*. *aureus* thereby exacerbating the bacterial pneumonia [2–4, 48]. Our observations are in line with these studies indicating that higher titers of bacteria can be seen during co-infection with norovirus.

MNV is known to cause apoptosis in RAW 264.7 cells, which is accompanied by rapid CPE as manifested by cell rounding, detachment, and floating of cells at 48 h post infection [23]. Our results indicated drastic reduction of MNV-induced CPE in cells infected with *S*. *enterica*, demonstrating that apoptosis could be blocked by the bacterium (Figs 3 and 4). MNV-induced apoptosis involves the activation of the intrinsic immune pathway and involvement of caspase-9 and caspase-3 effector molecules [23]. Our findings demonstrate that *Salmonella* infection altered the MNV response, preventing DNA fragmentation, PARP cleavage and blockade of caspase 9- and 3 cleavage events. *Salmonella* strains can be invasive intracellular pathogens, surviving in phagocytic vacuoles [8], that prevent the cells from undergoing apoptosis through the activation of cell survival pathways. *S*. Typhimurium has been shown to prevent camptothecin-induced apoptosis in HeLa cells and rat small intestinal epithelial cells by activating the PI3K/Akt pathway, which further prevents cytochrome C release and caspase-induced apoptosis [31, 32]. These activities have been attributed to the type III secretion system effector SopB, as the SopB mutant fails to prevent apoptosis [32]. Whether SopB is also involved in apoptosis prevention in *S*. Heidelberg remains to be determined; however, due to the similarity of the serotypes, it is probable. Similar mechanisms of preventing cell death and enhancing cell survival have been reported with other intracellular bacterial pathogens, including *Chlamydia* and *Neisseria*; where *Chlamydia trachomatis* inhibits staurosporine and UV-induced apoptosis by preventing BAX- and BAK-induced mitochondrial membrane permeabilization [49–51]. Further studies are underway to identify upstream effector molecules induced by *Salmonella* that might interact to block MNV-induced apoptosis.

Downregulation of apoptosis inducing genes in RAW 264.7 cells has been previously documented with several serotypes of *S*. *enterica* [30], including Heidelberg. *S*. Typhimurium upregulates necrotic pathways in macrophages when infected at a very high MOI, while downregulating apoptotic pathways [30, 52, 53]. Our results clearly indicate an inhibition of caspase-mediated apoptosis by *S*. Heidelberg. Apoptosis blockade by this bacterium might represent a survival strategy to establish persistent or chronic infections. MNV infection is highly cytopathic to RAW 264.7 cells, and the blockade of MNV induced apoptosis might signal the establishment of a prolonged infection by the bacterium. Further studies are needed to identify bacterial determinants that are responsible for inhibition of apoptosis in MNV infected cells.

The reduction of MNV replication in infected cells could also occur due to induction of cytokines that might promote an antiviral effect. Our results demonstrated the upregulation of IL6, IFNγ and TNFα in cells infected with the bacterium. Although previous studies have shown that IFNγ does not play a major role in controlling MNV infection ([54, 55], interferon stimulated gene ISG15 has been shown to function as an antiviral effector during MNV lifecycle[56]. This along with bacterial induction of IL6 and TNFα could have a synergistic effect in reducing MNV replication. Transcriptional profiling studies to identify specific subsets of upregulated genes that have antiviral effects and might contribute to this phenomenon are currently being investigated.

Overall, our study reports a novel mechanism, where alteration of host response at a molecular level, leads to reduction in MNV replication in *S*. *enterica* infected cells. Our observations suggest the possibility of enhanced bacterial replication and disease and/or establishment of persistent viral infection in an individual infected with both pathogens at the same time. Taken together, our study shows a mechanism where a bacterial pathogen modulates the host response and prevents virus adhesion, virus-induced cytopathicity and cell death. Further studies are needed to define the exact genetic determinants of the bacterium and the virus that contribute to the observed effects, as these may shed more light on the modification of the host by the bacteria and establishment of a prolonged infection.

## References

[1] Blyth CC, Webb SA, Kok J, Dwyer DE, van Hal SJ, Foo H, et al The impact of bacterial and viral co-infection in severe influenza. Influenza Other Respir Viruses. 2013;7(2):168–76. 10.1111/j.1750-2659.2012.00360.x . 22487223 PMC5006004

[2] McCullers JA. The co-pathogenesis of influenza viruses with bacteria in the lung. Nat Rev Microbiol. 2014;12(4):252–62. 10.1038/nrmicro3231 . 24590244

[3] Peltola VT, Boyd KL, McAuley JL, Rehg JE, McCullers JA. Bacterial sinusitis and otitis media following influenza virus infection in ferrets. Infect Immun. 2006;74(5):2562–7. 10.1128/IAI.74.5.2562-2567.2006 16622191 PMC1459735

[4] Smith AM, McCullers JA. Secondary bacterial infections in influenza virus infection pathogenesis. Curr Top Microbiol Immunol. 2014;385:327–56. 10.1007/82_2014_394 . 25027822 PMC7122299

[5] Iverson AR, Boyd KL, McAuley JL, Plano LR, Hart ME, McCullers JA. Influenza virus primes mice for pneumonia from Staphylococcus aureus. J Infect Dis. 2011;203(6):880–8. 10.1093/infdis/jiq113 21278211 PMC3071123

[6] Karst SM, Wobus CE, Goodfellow IG, Green KY, Virgin HW. Advances in norovirus biology. Cell Host Microbe. 2014;15(6):668–80. 10.1016/j.chom.2014.05.015 24922570 PMC4113907

[7] Foley SL, Nayak R, Hanning IB, Johnson TJ, Han J, Ricke SC. Population dynamics of Salmonella enterica serotypes in commercial egg and poultry production. Appl Environ Microbiol. 2011;77(13):4273–9. 10.1128/AEM.00598-11 21571882 PMC3127710

[8] Foley SL, Johnson TJ, Ricke SC, Nayak R, Danzeisen J. Salmonella pathogenicity and host adaptation in chicken-associated serovars. Microbiol Mol Biol Rev. 2013;77(4):582–607. 10.1128/MMBR.00015-13 24296573 PMC3973385

[9] Gonzalez-Galan V, Sánchez-Fauqier A, Obando I, Montero V, Fernandez M, Torres MJ, et al High prevalence of community-acquired norovirus gastroenteritis among hospitalized children: a prospective study. Clin Microbiol Infect. 2011;17(12):1895–9. 10.1111/j.1469-0691.2011.03506.x . 21848976

[10] Patel MM, Widdowson MA, Glass RI, Akazawa K, Vinjé J, Parashar UD. Systematic literature review of role of noroviruses in sporadic gastroenteritis. Emerg Infect Dis. 2008;14(8):1224–31. 10.3201/eid1408.071114 18680645 PMC2600393

[11] Lanata CF, Fischer-Walker CL, Olascoaga AC, Torres CX, Aryee MJ, Black RE, et al Global causes of diarrheal disease mortality in children <5 years of age: a systematic review. PLoS One. 2013;8(9):e72788 10.1371/journal.pone.0072788 24023773 PMC3762858

[12] Ahmed SM, Hall AJ, Robinson AE, Verhoef L, Premkumar P, Parashar UD, et al Global prevalence of norovirus in cases of gastroenteritis: a systematic review and meta-analysis. The Lancet Infectious diseases. 2014;14(8):725–30. 10.1016/S1473-3099(14)70767-4 . 24981041 PMC8006533

[13] Rha B, Burrer S, Park S, Trivedi T, Parashar UD, Lopman BA. Emergency department visit data for rapid detection and monitoring of norovirus activity, United States. Emerg Infect Dis. 2013;19(8):1214–21. 10.3201/eid1908.130483 23876432 PMC3739513

[14] Trivedi TK, Desai R, Hall AJ, Patel M, Parashar UD, Lopman BA. Clinical characteristics of norovirus-associated deaths: a systematic literature review. Am J Infect Control. 2013;41(7):654–7. 10.1016/j.ajic.2012.08.002 . 23266383

[15] Yen C, Wikswo ME, Lopman BA, Vinje J, Parashar UD, Hall AJ. Impact of an emergent norovirus variant in 2009 on norovirus outbreak activity in the United States. Clin Infect Dis. 2011;53(6):568–71. 10.1093/cid/cir478 . 21832262

[16] Debbink K, Lindesmith LC, Baric RS. The state of norovirus vaccines. Clin Infect Dis. 2014;58(12):1746–52. 10.1093/cid/ciu120 24585561 PMC4036685

[17] Aliabadi N, Lopman BA, Parashar UD, Hall AJ. Progress toward norovirus vaccines: considerations for further development and implementation in potential target populations. Expert Rev Vaccines. 2015;14(9):1241–53. 10.1586/14760584.2015.1073110 . 26224658 PMC5798453

[18] Green SM, Dingle KE, Lambden PR, Caul EO, Ashley CR, Clarke IN. Human enteric Caliciviridae: a new prevalent small round-structured virus group defined by RNA-dependent RNA polymerase and capsid diversity. J Gen Virol. 1994;75 (Pt 8):1883–8. . 8046390 10.1099/0022-1317-75-8-1883

[19] LoBue AD, Lindesmith L, Yount B, Harrington PR, Thompson JM, Johnston RE, et al Multivalent norovirus vaccines induce strong mucosal and systemic blocking antibodies against multiple strains. Vaccine. 2006;24(24):5220–34. 10.1016/j.vaccine.2006.03.080 . 16650512

[20] Gonzalez MD, Langley LC, Buchan BW, Faron ML, Maier M, Templeton K, et al Multi-Center Evaluation of the Xpert Norovirus Assay for Detection of Norovirus GI and GII in Fecal Specimens. J Clin Microbiol. 2015 10.1128/JCM.02361-15 . 26560532 PMC4702714

[21] Lindesmith LC, Donaldson EF, Baric RS. Norovirus GII.4 strain antigenic variation. J Virol. 2011;85(1):231–42. Epub 2010/10/29. doi: JVI.01364-10 [pii] 10.1128/JVI.01364-10 20980508 PMC3014165

[22] Vinje J. Advances in Laboratory Methods for Detection and Typing of Norovirus. Journal of clinical microbiology. 2015;53(2):373–81. 10.1128/JCM.01535-14 24989606 PMC4298492

[23] Bok K, Prikhodko VG, Green KY, Sosnovtsev SV. Apoptosis in murine norovirus-infected RAW264.7 cells is associated with downregulation of survivin. J Virol. 2009;83(8):3647–56. 10.1128/JVI.02028-08 19211757 PMC2663291

[24] Wobus CE, Thackray LB, Virgin HWt. Murine norovirus: a model system to study norovirus biology and pathogenesis. Journal of virology. 2006;80(11):5104–12. 10.1128/JVI.02346-05 16698991 PMC1472167

[25] Hwang S, Alhatlani B, Arias A, Caddy SL, Christodoulou C, Cunha JB, et al Murine norovirus: propagation, quantification, and genetic manipulation. Curr Protoc Microbiol. 2014;33:15K.2.1–K.2.61. 10.1002/9780471729259.mc15k02s33 24789596 PMC4074558

[26] Jones MK, Watanabe M, Zhu S, Graves CL, Keyes LR, Grau KR, et al Enteric bacteria promote human and mouse norovirus infection of B cells. Science. 2014;346(6210):755–9. 10.1126/science.1257147 25378626 PMC4401463

[27] Scallan E, Crim SM, Runkle A, Henao OL, Mahon BE, Hoekstra RM, et al Bacterial Enteric Infections Among Older Adults in the United States: Foodborne Diseases Active Surveillance Network, 1996–2012. Foodborne Pathog Dis. 2015;12(6):492–9. 10.1089/fpd.2014.1915 . 26067228 PMC4630801

[28] Newman KL, Leon JS, Rebolledo PA, Scallan E. The impact of socioeconomic status on foodborne illness in high-income countries: a systematic review. Epidemiol Infect. 2015:1–13. 10.1017/S0950268814003847 . 25600652 PMC4508232

[29] Andino A, Hanning I. Salmonella enterica: survival, colonization, and virulence differences among serovars. ScientificWorldJournal. 2015;2015:520179 10.1155/2015/520179 25664339 PMC4310208

[30] Gokulan K, Khare S, Rooney AW, Han J, Lynne AM, Foley SL. Impact of plasmids, including those encodingVirB4/D4 type IV secretion systems, on Salmonella enterica serovar Heidelberg virulence in macrophages and epithelial cells. PLoS One. 2013;8(10):e77866 10.1371/journal.pone.0077866 24098597 PMC3789690

[31] Knodler LA, Finlay BB. Salmonella and apoptosis: to live or let die? Microbes Infect. 2001;3(14–15):1321–6. . 11755421 10.1016/s1286-4579(01)01493-9

[32] Knodler LA, Finlay BB, Steele-Mortimer O. The Salmonella effector protein SopB protects epithelial cells from apoptosis by sustained activation of Akt. J Biol Chem. 2005;280(10):9058–64. 10.1074/jbc.M412588200 . 15642738

[33] Gonzalez-Hernandez MB, Bragazzi Cunha J, Wobus CE. Plaque assay for murine norovirus. J Vis Exp. 2012;(66):e4297 10.3791/4297 22951568 PMC3487293

[34] Hewitt J, Rivera-Aban M, Greening GE. Evaluation of murine norovirus as a surrogate for human norovirus and hepatitis A virus in heat inactivation studies. J Appl Microbiol. 2009;107(1):65–71. 10.1111/j.1365-2672.2009.04179.x . 19298511 PMC7197740

[35] Kolawole AO, Xia C, Li M, Gamez M, Yu C, Rippinger CM, et al Newly isolated mAbs broaden the neutralizing epitope in murine norovirus. J Gen Virol. 2014;95(Pt 9):1958–68. 10.1099/vir.0.066753-0 24899153 PMC4135088

[36] Han S, Sung KH, Yim D, Lee S, Cho K, Lee CK, et al Activation of murine macrophage cell line RAW 264.7 by Korean propolis. Arch Pharm Res. 2002;25(6):895–902. . 12510845 10.1007/BF02977011

[37] Soldani C, Lazzè MC, Bottone MG, Tognon G, Biggiogera M, Pellicciari CE, et al Poly(ADP-ribose) polymerase cleavage during apoptosis: when and where? Exp Cell Res. 2001;269(2):193–201. 10.1006/excr.2001.5293 . 11570811

[38] Elmore S. Apoptosis: a review of programmed cell death. Toxicol Pathol. 2007;35(4):495–516. 10.1080/01926230701320337 17562483 PMC2117903

[39] Lemasters JJ, Qian T, Elmore SP, Trost LC, Nishimura Y, Herman B, et al Confocal microscopy of the mitochondrial permeability transition in necrotic cell killing, apoptosis and autophagy. Biofactors. 1998;8(3–4):283–5. . 9914830 10.1002/biof.5520080316

[40] Lemasters JJ, Nieminen AL, Qian T, Trost LC, Elmore SP, Nishimura Y, et al The mitochondrial permeability transition in cell death: a common mechanism in necrosis, apoptosis and autophagy. Biochim Biophys Acta. 1998;1366(1–2):177–96. . 9714796 10.1016/s0005-2728(98)00112-1

[41] Lemasters JJ, Qian T, He L, Kim JS, Elmore SP, Cascio WE, et al Role of mitochondrial inner membrane permeabilization in necrotic cell death, apoptosis, and autophagy. Antioxid Redox Signal. 2002;4(5):769–81. 10.1089/152308602760598918 . 12470504

[42] Metkar SS, Wang B, Catalan E, Anderluh G, Gilbert RJ, Pardo J, et al Perforin rapidly induces plasma membrane phospholipid flip-flop. PLoS One. 2011;6(9):e24286 10.1371/journal.pone.0024286 21931672 PMC3171411

[43] Chen YC, Wang SY, King CC. Bacterial lipopolysaccharide inhibits dengue virus infection of primary human monocytes/macrophages by blockade of virus entry via a CD14-dependent mechanism. J Virol. 1999;73(4):2650–7. 10074110 10.1128/jvi.73.4.2650-2657.1999 PMC104020

[44] Krakauer T, Peters CJ. Lipopolysaccharide inhibits the production of lymphocytic choriomeningitis virus in a human monocytic cell line. J Gen Virol. 1993;74 (Pt 8):1653–6. . 8345356 10.1099/0022-1317-74-8-1653

[45] Bernstein MS, Tong-Starksen SE, Locksley RM. Activation of human monocyte—derived macrophages with lipopolysaccharide decreases human immunodeficiency virus replication in vitro at the level of gene expression. J Clin Invest. 1991;88(2):540–5. 10.1172/JCI115337 1907615 PMC295381

[46] Bagasra O, Wright SD, Seshamma T, Oakes JW, Pomerantz RJ. CD14 is involved in control of human immunodeficiency virus type 1 expression in latently infected cells by lipopolysaccharide. Proc Natl Acad Sci U S A. 1992;89(14):6285–9. 1378624 10.1073/pnas.89.14.6285 PMC49485

[47] Weinberger DM, Klugman KP, Steiner CA, Simonsen L, Viboud C. Association between respiratory syncytial virus activity and pneumococcal disease in infants: a time series analysis of US hospitalization data. PLoS Med. 2015;12(1):e1001776 10.1371/journal.pmed.1001776 25562317 PMC4285401

[48] Lahti E, Peltola V, Virkki R, Ruuskanen O. Influenza pneumonia. Pediatr Infect Dis J. 2006;25(2):160–4. 10.1097/01.inf.0000199265.90299.26 . 16462295

[49] Miyairi I, Byrne GI. Chlamydia and programmed cell death. Curr Opin Microbiol. 2006;9(1):102–8. 10.1016/j.mib.2005.12.004 . 16406838

[50] Zhong Y, Weininger M, Pirbhai M, Dong F, Zhong G. Inhibition of staurosporine-induced activation of the proapoptotic multidomain Bcl-2 proteins Bax and Bak by three invasive chlamydial species. J Infect. 2006;53(6):408–14. 10.1016/j.jinf.2005.12.028 . 16490255

[51] Häcker G, Kirschnek S, Fischer SF. Apoptosis in infectious disease: how bacteria interfere with the apoptotic apparatus. Med Microbiol Immunol. 2006;195(1):11–9. 10.1007/s00430-005-0239-4 . 16086183

[52] Knodler LA, Crowley SM, Sham HP, Yang H, Wrande M, Ma C, et al Noncanonical inflammasome activation of caspase-4/caspase-11 mediates epithelial defenses against enteric bacterial pathogens. Cell Host Microbe. 2014;16(2):249–56. 10.1016/j.chom.2014.07.002 25121752 PMC4157630

[53] Knodler LA. Salmonella enterica: living a double life in epithelial cells. Curr Opin Microbiol. 2015;23:23–31. 10.1016/j.mib.2014.10.010 . 25461569

[54] Wobus CE, Karst SM, Thackray LB, Chang KO, Sosnovtsev SV, Belliot G, et al Replication of Norovirus in cell culture reveals a tropism for dendritic cells and macrophages. PLoS Biol. 2004;2(12):e432 10.1371/journal.pbio.0020432 15562321 PMC532393

[55] Chachu KA, LoBue AD, Strong DW, Baric RS, Virgin HW. Immune mechanisms responsible for vaccination against and clearance of mucosal and lymphatic norovirus infection. PLoS Pathog. 2008;4(12):e1000236 10.1371/journal.ppat.1000236 19079577 PMC2587711

[56] Rodriguez MR, Monte K, Thackray LB, Lenschow DJ. ISG15 functions as an interferon-mediated antiviral effector early in the murine norovirus life cycle. J Virol. 2014;88(16):9277–86. 10.1128/JVI.01422-14 24899198 PMC4136287

